# Surgery

**Published:** 1893-09

**Authors:** W. J. Penny


### SURGERY.

It seems now to be very generally conceded that when a cancer of the rectum is diagnosed, the operative treatment that offers the most satisfactory result, practically resolves itself into two procedures, colotomy and excision. It must not be forgotten, however, that the field of usefulness of excision for carcinoma of the rectum is very limited; but in well-selected cases much good may be done by lessening pain, prolonging life, or even at times effecting a cure. Cripps and most English writers place the proportion of cases suitable for excision at from 15 to 20 per cent.; the Germans, on the other hand, subject to these radical measures 75 per cent.

The methods usually employed may be divided into three classes: the perineal, the sacral, and the combined perineal and sacral. The perineal is employed mostly for growths about the anus; the sacral is most useful when a circumscribed growth is too high for the perineal plan and too low for laparotomy. The combined method makes it possible to either resect long pieces of rectum or to amputate long portions which would otherwise have been inoperable on account of their distance from the perineum. Some authorities recommend that a previous colotomy should be done.

To indicate operation, Cripps holds that the offending mass must be freely movable in all directions, and there should be a fair chance of completely removing the diseased structures. The most promising cases are those that resemble an ulcer, are limited to one side of the gut, have the upper border well defined, and healthy mucous membrane beyond the growth which can be felt. Allingham looks with disfavour on attacking a growth four inches from the anal orifice; Treves opposes the procedure when the peritoneum is opened, or portions of the bladder, vagina, or prostate are removed. The Germans go higher, and open the peritoneal cavity with greater impunity.

McCosh[1] gives a table of 439 cases operated on by well-known surgeons: 84, or 19.1 per cent., deaths are attributable to the procedure.

As regards prognosis, 375 cases are quoted. Considering an immunity for four years as a cure, McCosh claims that these can be reckoned at 11 per cent.

The comparative values of the different operations for rectal cancer, palliative and radical, may be gathered from the published records of various surgeons. Préchaud's tables,[2] made up from his own cases and the records of several other operators, give 149 cases of rectal cancer excisions, of which there were 36 operative deaths—a mortality of rather over 24 per cent.; 103 operative cures, but with immediate relapse in 3; 69 relapsed after one year, 15 relapsed after two years, 2 after three years, 5 after four years, 5 after five years. Hence from the above we must assume that five years after operation none but six could be accounted for, or about 4 per cent. of all.

Charron gives us a table of results in 139 cases of colotomy: of these, 8 were missing and could not be accounted for, 54 died within two months, 65 lived from two months to one year, 5 lived from twelve to eighteen months, 3 lived from one year to two years, 6 lived two years or more.

Dr. Thomas H. Manley, from the above tables, considers there is little difference between the operations as far as the prolongation of life is concerned; and the real question to determine, in a case of cancer of the rectum, is, not what operation will eradicate the malady, for that is clearly out of the question, but

[1] *Amer. Pract. and News*, June 17, 1893.

[2] *Journ. Amer. Med. Assoc.*, Ap. 8, 1893.

rather what will afford a temporary cure, and give the patient the greatest amount of mental quiet and bodily comfort, so that when the end comes it may be painless, as we know is commonly the case when death is due to cancer of the internal organs.

A further series of cases of rectal excision is reported by Mr. Harrison Cripps.[1] Of upwards of 400 cases examined by the author in fifteen years, in about one-half any operative treatment was advised against. Of the remainder, 114 were operated upon—38 by excision, 76 by colotomy. Of the 38 cases of excision, 3 died from the operation, 35 recovered; of these that recovered the subsequent history is as follows:

In 7 no reliable history was found
10 recurrence within one year
5 recurrence between first and third year
1 died a year later without recurrence
1 no recurrence after eighteen months

| | | |
|---|---|---|
| 10 no recurrence | 3 | under 1 year |
| | 1 | after 2 years |
| | 1 | " 3 " |
| | 2 | " 4 " |
| | 1 | " 5 " |
| | 2 | " 6 " |
| | 1 | " 12 " |

In seven of these cases, over three years had passed without relapse, so that they are considered as cures. This series of cases shows a better percentage of cures, nearly 18.5 per cent. In one of the cases the growth was adherent to the coccyx and lower sacral bone; and in another, a fungous mass protruded into the vagina; in another, the growth was five inches from the anus. In three of these a second operation had to be performed; and in one a slight nodule was removed on a third occasion.

As regards the comfort after excision, incontinence appears to be the rule immediately after operation; but as the wound heals, control is gained. Retention of the fæces is possible in many instances, even after the entire sphincter is cut away. The contraction of the wound, which is very troublesome after the first few weeks, can be overcome by the regular use of a bougie; this should be continued for a year or even longer; after this, the tendency to contract appears to be gradually lost, and gives little trouble after the second year.

Professor Bruns reports[2] four cases in which he operated successfully for dislocation of one of the semilunar cartilages of the knee; and from literature collects others to the number of 43. The dislocation affected the internal cartilage 27 times out of the 40 in which the point was specified, contrary to the opinion usually held. The dislocation is usually partial, and

[1] See *Annals of Surgery*, Mar. and Ap., 1893.
[2] See *Inter. Med. Mag.*, Mar., 1893.

generally concerns the anterior portion; although the posterior end, or even the middle third of the cartilage, has been found torn from its attachments. A rarer form of injury is that in which the cartilage is wholly or partially torn across, or even crushed into a shapeless mass of fibres at one end. The dislocation follows almost always upon a traumatism; but in some cases a gradual loosening of the attachments seems to take place, a sort of spontaneous dislocation following. But Bruns emphatically denies that it is liable to follow over-distension of the capsule, as in hydrops; in fact, he considers short and strong ligamentous structures as necessary for the displacement, which, in his opinion, occurs in forced rotation with the knee flexed. The diagnosis should be easy, by reason of the peculiar locking of the joint and the presence of the displaced meniscus close to the joint-cleft.

In some cases the cartilage cannot be felt, but the joint-cleft is often found widened and more evident than normal, and somewhat tender. Treatment of the fresh dislocation would consist of immobilisation for some four or six weeks; but even then it will not, as a rule, bring firm adhesions. Where the dislocation has become habitual, only operation can serve: and here it seems to matter little whether the displaced part of the cartilage be resected, or returned and sutured in place; for the functional results seem to be perfect in either method, even where the cases have been followed for years. Twenty-four cases were treated by extirpation, the rest by suture, all recovering.

Dr. Braquehaye[1] has collected sixteen cases of these very rare luxations, and added one new case. On the basis of his studies he reaches the following conclusions. The luxation may be external or internal, corresponding to the luxated meniscus, and anteriorly or posteriorly from the lateral ligaments. The luxation in an external and anterior direction is the most frequent; inward and posterior most rare. The luxation occurs only in cases of flexion of the knee, the legs being separated. The direct causes include all traumatisms that stretch or tear the lateral ligaments; and, further, certain movements of the joint producing relaxation of the ligaments, as for instance rising from a stooping position.

The symptoms of the injury consist at first of an audible cracking, accompanied by a violent pain. When the luxation is complete, extension is impossible, and the patient is unable to place the foot on the ground. Usually he accomplishes reduction himself; but the same appearances recur, flexion and tension of the extremity being repeated. At the side of the patella a small, hard, flattened body is clearly prominent; an effusion into the joint does not usually occur immediately after the accident, but more frequently later on. The symptoms of the luxation backwards are very similar to those anteriorly. If

[1] See *Annals of Surgery*, Jan., 1893.

the prominence is not clearly felt, the diagnosis may often be obscure.

Cases of complete dislocation of the cartilage are, Dr. Braquehaye says, very rare; and cases of partial displacement in which the symptom of locking of the joint occurs, are not very common. There is a class of cases, however, in which I believe some slight displacement occurs, which are not at all uncommon. The symptoms are those of a severe strain, with great pain and tenderness over the attachment of the cartilage to the tibia, slight inflammatory œdema there, and a good deal of effusion into the joint. The treatment in these cases is the same as that recommended by Professor Bruns—immobilisation for some weeks, with subsequent support of the knee for a time by some form of knee-cap or bandage. A recurrence of the symptoms is not unlikely to occur if the joint be again subjected to a strain; and occasionally a case is met with in which, after one or two recurrences, the displacement is sufficiently marked for the symptom of locking to occur.

Professor König, in a paper[1] on "The Modern Treatment of Tuberculous Joint-Disease," states that only one-fifth of the cases of tuberculosis of the joints are without lesions elsewhere, and therefore any treatment limited to the joint cannot be called radical. Many of our methods of treatment, moreover, merely cause the disappearance of the symptoms, by the encapsulation of the foci of disease in the joint, not by their removal; and yet the results obtained in this way, if the function of the joint is preserved, are better than those obtained by more radical measures, although there may be danger of recurrence. There is then no truly radical treatment. Very early resections have fallen entirely into disuse. Early cases are to be treated by immobilisation with some form of apparatus. If this fails, iodoform or other cicatrising injections are to be made; and only in cases of continued spread of the disease is resection to be resorted to. An approximation of the proportions in these different classes is to be obtained by a study of the hip-joint cases in Professor König's clinic. In about 400 of these cases, 150 recovered after treatment for one or two years by extension or plaster-of-Paris apparatus, 50 with injections of iodoform and incision of abscesses, and 210 were resected. The existence of abscesses and profusely discharging fistulæ is no contra-indication to the iodoform treatment; in fact many of these cases recover with greater rapidity than those which are apparently milder. The least promising cases for this method are those with severe bony lesions; and if one could recognise these at once, it would simplify the matter very much: but at present we cannot distinguish them in the beginning, consequently a trial of the injection method should be made; and if there is no

[1] See *Inter. Med. Mag.*, Mar., 1893.

improvement after four or five injections, operation will be necessary.

In the selection of the form of operation, simple extirpation of the capsule, without removal of bone and with very little curetting, should be reserved for children. Adults require removal of the joint-ends as well as the extirpation of the capsule, else some bone lesion may be overlooked. Amputation will still be necessary in some cases, especially when the disease is very extensive, when there is septic infection, or when there is disease of the kidneys or liver.

Concerning mercurial treatment, Dr. Charles Mauriac[1] believes that mercury, in common with other drugs which at the same time are poisonous and of therapeutic value, must be so used that the different degrees of curative action shall be exhibited according to the circumstances of each case; and yet toxic effects which may be even antagonistic to its therapeutic action may be avoided. The curative principle of the remedy does not change, it matters not by what means it is introduced into the system, nor indeed in what form. The question of dose is important, and the differences in results arise only from the rapidity with which the system can be mercurialised. The rapidity of the curative action does not imply that it shall be of long duration, or that it may have some preventive power if it be slow and gradual.

In commencing treatment, it is proper to place the patient upon a diet which shall aid the absorption of the remedy, and to limit to some extent the nutritive functions. From time to time, purgation is proper; we must supervise the diet, limiting it in quantity, and choosing it according to the temperament of the individual.

In a considerable number of patients, particularly in women, the question of tonics, reconstituents, and substantial diet will arise. The mouth and teeth should be attended to before treatment is commenced, and the digestive functions carefully examined. Above all things, the functions of the kidney should be attended to, for it is by these organs the remedy is eliminated.

In all cases the treatment must be adapted to the individual, and the particular condition of that individual at different times.

Mr. Jonathan Hutchinson writes: "I may here avow that, after plentiful opportunities for the observation of the different methods, I have adopted the practice of keeping the skin methods in reserve for exceptional cases, and that under all ordinary circumstances I administer the remedy by the mouth. One simple rule appears to be the key to success. It is to give small doses more or less frequently repeated, and never large

[1] See *Amer. Journ. Med. Sci.*, July, 1893.

ones. Hydrargyrum cum cretâ is, perhaps, the most constant and least variable of all preparations. It may be made into pills of one grain, in combination with one grain of Dover's powder, if necessary, and of these the patient may take one every six, four, three, or even two hours, according to circumstances. Usually, one pill four times a day will suffice to clear away a chancre or a secondary eruption as rapidly and as completely as can be wished; but if at any period within six months the mercury be suspended, within a few weeks of the suspension a rash may show itself. During a mercurial course, fruit, green vegetables, coffee, all aperients, and for the most part all stimulants, should be forbidden; and it is better to avoid smoking."[1]

Mr. Hutchinson refers [2] to cases in which patients with syphilis, who have been successfully treated by abortive methods, come to entertain a suspicion that after all they have never had the disease. Those who practice the abortive method must look to encounter not infrequently very hesitating gratitude on the part of their patients. The popular idea of syphilis is that it is necessarily attended by a loathsome eruption and great loss of health. When the chancres pass away and nothing whatever follows, the patient begins to suspect that there has been an error. He cannot believe that a few boxes of little pills have really prevented so much, and as frequently becomes suspicious of a blunder as grateful for skill. This unfortunate result is especially likely to occur if the surgeon be not one engaged in special practice. Mr. Hutchinson therefore advises all who use this method to explain fully that it is designed to prevent all consequences, and that probably with the disappearance of the chancre there will be an end of the matter.

In future years the abortive treatment will also render the recognition of a syphilitic history, in cases of remote tertiary disease, a matter of much more difficulty than at present. We now, in taking a patient's history, rely much on the previous occurrence of what we know as complete syphilis, meaning by that expression a chancre followed by rash and sore throat. In future, very few cases will have this complete record; almost all will tell us: "I once had a sore which Mr. —— said was a chancre; but it was soon well, and for my own part I do not believe it was one."

The great difficulty I have experienced, especially among hospital patients, is to induce them to continue the remedy long enough. They are apt to discontinue their attendance as soon as the symptoms for which they presented themselves disappear, and not infrequently they come up a short time afterwards with a recurrence, or some fresh manifestation of the disease, due, I believe, to an insufficiently long course of the remedy.

W. J. PENNY.

[1] *Syphilis*, 1887, p. 51. [2] *Arch. Surg.*, Jan., 1893, p. 244.

